# Omega-3 Fatty Acid Enriched Chevon (Goat Meat) Lowers Plasma Cholesterol Levels and Alters Gene Expressions in Rats

**DOI:** 10.1155/2014/749341

**Published:** 2014-02-25

**Authors:** Mahdi Ebrahimi, Mohamed Ali Rajion, Goh Yong Meng, Abdoreza Soleimani Farjam

**Affiliations:** ^1^Department of Veterinary Preclinical Sciences, Faculty of Veterinary Medicine, Universiti Putra Malaysia (UPM), 43400 Serdang, Selangor, Malaysia; ^2^Institute of Tropical Agriculture, Universiti Putra Malaysia (UPM), 43400 Serdang, Selangor, Malaysia

## Abstract

In this study, control chevon (goat meat) and omega-3 fatty acid enriched chevon were obtained from goats fed a 50% oil palm frond diet and commercial goat concentrate for 100 days, respectively. Goats fed the 50% oil palm frond diet contained high amounts of *α*-linolenic acid (ALA) in their meat compared to goats fed the control diet. The chevon was then used to prepare two types of pellets (control or enriched chevon) that were then fed to twenty-male-four-month-old *Sprague-Dawley* rats (*n* = 10 in each group) for 12 weeks to evaluate their effects on plasma cholesterol levels, tissue fatty acids, and gene expression. There was a significant increase in ALA and docosahexaenoic acid (DHA) in the muscle tissues and liver of the rats fed the enriched chevon compared with the control group. Plasma cholesterol also decreased (*P* < 0.05) in rats fed the enriched chevon compared to the control group. The rat pellets containing enriched chevon significantly upregulated the key transcription factor PPAR-*γ* and downregulated SREBP-1c expression relative to the control group. The results showed that the omega-3 fatty acid enriched chevon increased the omega-3 fatty acids in the rat tissues and altered PPAR-*γ* and SREBP-1c genes expression.

## 1. Introduction

Researchers had succeeded in feeding goats with linseed oil [1] and polyphenol rich oil palm frond (OPF) diets which reduced microbial biohydrogenation in the rumen [2] and producing chevon (goat meat) containing increased levels of *α*-linolenic acid (ALA, C18:3n-3). However, it remained to be determined whether consuming this “modified” chevon containing the higher levels of *α*-linolenic acid would also, in turn, increase the levels of the omega-3 fatty acids (FAs) in the tissues of the consumer, which could produce measurable positive health benefits.

The *α*-linolenic acid, a nutritionally essential fatty acid that must be obtained through the diet, can be converted in the vertebrate liver or brain to docosahexaenoic acid (DHA, C22:6n-3) [3] via serial steps of desaturation and elongation with final peroxisomal chain shortening [3], by means of desaturases and elongases [4, 5]. The *α*-linolenic acid can be converted to Eecosapentaenoic acid (EPA) which can be converted to DHA [6]. The best available source of long-chain omega-3 FAs is marine products such as fish which many people avoid eating [7]. Therefore, if consuming foods high in the shorter-chain omega-3 FAs, such as flaxseed oil or enriched omega-3 animal products, gives a positive heart health advantage, it would improve the public health [8]. Making foods rich in ALA can also help increase the level of this FA in the diet.

Many studies also showed that dietary omega-3 FAs work as biological regulators with several physiological and biological roles. They form part of the fundamental cell membrane, modify gene expression, and act as signaling molecules [9–11]. Many researchers have demonstrated that omega-3 FAs can enhance the lipid metabolism by lowering plasma triglycerides. Little is known if the chevon fortified with omega-3 FAs can produce these effects. Moreover, the molecular mechanisms underlying the effects of these dietary nutrients have not been well studied. We hypothesize that the omega-3 enriched chevon can improve lipid metabolism through regulation of polyunsaturated FA (PUFA) metabolism, thereby altering transcription factors like peroxisome proliferator-activated receptors (PPARs) and their downstream genes like Sterol Regulatory Element-Binding Proteins (SREBPs). In this study, the effects of feeding pellets made from omega-3 enriched chevon on the plasma cholesterol levels and tissue FA profiles in rats, as bioindicators, were investigated. Additionally, the effects of these diets on the hepatic expression of transcription factors and genes involved in FA metabolism were also evaluated.

## 2. Materials and Methods

### 2.1. Animals and Ethical Considerations

All the procedures and techniques related to the use, care of animals for research, and the experimental design were undertaken following the guidelines of the Research Policy of the Universiti Putra Malaysia on animal ethics.

### 2.2. Enriched Chevon

Initially, 16 goats with an initial weight of 22.81 ± 0.97 kg were fed with either a diet consisting of 50% oil palm frond (OPF) on a dry weight basis with 50% concentrate or a control diet containing 100% concentrate. The goats were housed in individual wooden pens with raised flooring and fed for 100 days. After slaughter, the entire meat from the carcass was removed and stored at −20°C.

### 2.3. Experimental Design

This study was conducted with two dietary treatment groups, namely, an omega-3 enriched chevon diet made of 80% meat from the OPF-fed goats + 20% commercial rat chow power and a control diet made up of 80% meat from control-fed goats + 20% commercial rat chow powder (Table 1). The control and enriched chevon were minced with a meat grinder. The mixture of chevon and rat chow powder was made into dough and pelleted using a modified meat grinder. The meat grinder was fitted with a compression barrel measuring 24 cm long and 1.7 cm to yield pellets that are compact enough. The pellets were then oven-dried at 50–55°C for 48 h until acceptable dryness. The pellets were packed and sealed in plastic buckets in packs of 20 kg and stored at −20°C. Twenty-four-month-old male* Sprague-Dawley* rats were fed (*n* = 10 in each group) either meat pellets prepared from the control or the omega-3 enriched chevon for 12 weeks. The rats received food and water *ad libitum.* They were kept individually in polyethylene cages. Wood shaving was used as bedding and the mesh cover was stainless steel. The room was well ventilated and the ambient temperature was between about 22 and 25°C with a 12-hour light and dark cycle.

### 2.4. Fatty Acid Analysis

After 12 weeks from the commencement of the study, the rats were anesthetized by intraperitoneal injection of ketamine-xylazine (Ketamine 50 mg/kg and Xylazine 10 mg/kg) as the anesthetic agent. The muscle tissues were sampled from the gluteal muscle. The heart and liver were collected and snap-frozen in liquid nitrogen. The samples were stored at −80°C until further analysis. The total FA was extracted from the experimental feeds, liver, and heart tissues by the method of Folch et al. [12], modified by Rajion et al. [13], and described by Ebrahimi et al. [2], using chloroform: methanol 2 : 1 (v/v) containing butylated hydroxytoluene to prevent oxidation during sample preparation. An internal standard, heneicosanoic acid (C21 : 0) (Sigma Chemical Co., St. Louis, MO, USA), was added to each sample prior to transmethylation to determine the individual FA concentrations within the samples. The extracted fatty acids were transmethylated to their fatty acid methyl esters (FAME) using 0.66 N KOH in methanol and 14% methanolic boron trifluoride (BF_3_) (Sigma Chemical Co. St. Louis, MO, USA) according to the methods by AOAC [14].

### 2.5. Fatty Acid Analysis

The FAME was separated by gas liquid chromatography on an Agilent 7890A GC system (Agilent, Palo Alto, CA, USA) using a 100 m × 0.25 mm ID (0.20 µm film thickness) Supelco SP-2560 capillary column (Supelco, Inc., Bellefonte, PA, USA). One microliter of FAME was injected by an autosampler into the chromatograph and equipped with a flame ionization detector (FID). The carrier gas was He and the split ratio was 10 : 1 after injection of the FAME. The injector temperature was programmed at 250°C and the detector temperature was 300°C. The column temperature program initiated runs at 120°C held for 5 min, increased by 2°C/min up to 170°C, held at 170°C for 15 min, increased again by 5°C/min up to 200°C, held at 200°C for 5 min, increased again by 2°C/min to a final temperature of 235°C, and held for 10 min. The FA concentrations are expressed as g/100 g of sample. A reference standard (mix C4–C24 methyl esters; Sigma-Aldrich, Inc., St. Louis, MO, USA) and individual FAME, methyl palmitate, methyl stearate, methyl oleate, methyl linoleate, methyl linolenate, gamma linolenate, methyl arachidonate, methyl eicosapentaenoate, and methyl docosahexaenoate were used to determine recoveries and correction factors for the determination of individual FA composition.

### 2.6. Plasma Cholesterol Determinations

Blood samples were collected from the heart and the abdominal aorta of the rats as described by Escudero et al. [15] and centrifuged at 3000 rpm for 10 min to collect the plasma. The plasma was then analyzed for total cholesterol using analytical kits (Pointe Scientific Inc., MI, USA) and determined calorimetrically on a Hitachi 902 Automatic chemical analyzer (Roche International, Basel, Switzerland).

### 2.7. Tissue Collection and RNA Extraction and Purification

Immediately after sacrificing the rats the liver tissues were quickly excised and snap-frozen in liquid nitrogen and stored at −80°C until RNA extraction.

Total RNA was extracted from 100 mg of frozen liver tissue using the RNeasy lipid tissue mini kit (Cat. number 74804, Qiagen, Hilden, Germany) and DNase digestion was completed during RNA purification using the RNase-Free DNase set (Qiagen, Hilden, Germany) according to the manufacturer's instructions. Total RNA purity was determined by the 260/280 nm ratio of absorbance readings using NanoDrop ND-1000 UV-Vis Spectrophotometer (NanoDrop Technologies, Wilmington, DE, USA).

### 2.8. Complementary DNA Synthesis

Purified total RNA (1 µg) was reverse transcribed using a Quantitect reverse transcription kit (Qiagen, Hilden, Germany) in accordance with the manufacturer's recommended procedure.

### 2.9. Real-Time Polymerase Chain Reaction (PCR)

Real-time PCR was performed with the Bio-Rad CFX96 Touch (Bio-Rad Laboratories, Hercules, CA, USA) using optical grade plates using Quantifast SYBR green PCR kit (Cat. number 204054, Qiagen, Hilden, Germany). The sequences of primers are shown in Table 2.

The *β*-actin was used as the reference gene to normalize the tested genes. All primers were purchased through 1st BASE oligonucleotide synthesis (1st Base, Singapore). Each reaction (20 µL) contained 8.5 µL SYBR green PCR mix, 1 µL cDNA, 1 µL each of forward and reverse primers, and 8.5 µL RNase free water. Target genes were amplified through the following thermo cycling program: 95°C for 10′, 40 PCR cycles at 95°C for 30′′, 60°C for 20^′′,^ and 72°C for 20′′. Fluorescence was measured at every 15′′ to construct the melting curve. A real-time PCR was conducted for each primer pair in which cDNA samples were substituted with dH2O to verify that exogenous DNA was not present. Additionally, 1 µg of RNA isolated by the procedure described above was substituted for cDNA in a real-time PCR reaction to confirm that there were no genomic DNA contaminants in the RNA samples. Both negative controls showed no amplification after 40 cycles. Efficiency of amplification was determined for each primer pair using serial dilutions. The cycle numbers at which amplified DNA samples exceeded a computer generated fluorescence threshold level were normalized and compared to determine the relative gene expression. Higher cycle number values indicated lower initial concentrations of cDNA and thus lower levels of mRNA expression. Each sample was run in triplicate, and averaged triplicates were used to assign cycle threshold (CT) values. The ΔCT values were generated by subtracting experimental CT values from the CT values for *β*-actin targets amplified with each sample. The group with the highest mean ΔCT value (lowest gene expression) per amplified gene target was set to zero and the mean ΔCT values of the other groups were set relative to this calibrator (ΔΔCT). The ΔΔCT values were calculated as powers of 2 (−2ΔΔCT), to account for the exponential doubling of the PCR.

### 2.10. Statistical Analysis

Results were analyzed using analysis of variance (ANOVA) with the different dietary treatments as the main effects. Body weights, FA data, plasma cholesterol, and all the gene expression data were analyzed by one-way ANOVA, using the MIXED procedure of the SAS software package, version 9.1 (SAS Inst. Inc., Cary, NC, USA). The statistical models used the following equation:

(1) $$\begin{array}{c} Yijk=µ+Ti+Fk+eijk, \end{array}$$

where *µ* was the overall mean, *T* was the different dietary chevon, *F* was the rat effect, and *e* was the residual error. The random effect was the rats. Means were separated using the “PDIFF” option of the “least-squares means (LSMEANS)” statement of the MIXED procedure. The data were checked for normality using the UNIVARIATE procedure of SAS software and the results in the Tables are presented as means ± standard error of the mean. Differences in a *P* value of <0.05 were considered to be significant.

## 3. Results

No animals died throughout the study. There were no significant differences in the macronutrient composition of the exprimental feed (Table 1). The total feed consumption for the CON and enriched chevon group was 2850.96 and 2927.40 g, respectively, which was not significantly (*P* > 0.05) different after 12 weeks of feeding trial. The body weight of the rats in the omega-3 enriched chevon group (375 g) was not significantly different (*P* > 0.05) from that of the CON group (362 g).

### 3.1. Dietary Fatty Acids

Table 3 shows the FA concentration of the experimental diets. There was a statistically significant (*P* < 0.05) higher amount of ALA, eicosapentaenoic acid (EPA), docosapentaenoic acid (DPA), and docosahexaenoic acid (DHA) in the omega-3 enriched chevon diet when compared to the CON diet.

### 3.2. Plasma Cholesterol Levels

Rats fed the omega-3 enriched chevon diet had significantly (*P* < 0.05) lower plasma cholesterol levels (79 ± 2.1 mg/dL) compared to rats fed on the CON diet (101 ± 5.3 mg/dL) after 12 weeks of feeding trial. However the level of the omega-3 enriched chevon group was not significantly different (*P* > 0.05) to the baseline plasma cholesterol levels for all the rats which was 55.29 ± 4.12 mg/dL.

### 3.3. Rat Muscle Tissue, Liver, and Heart Fatty Acids

As shown in Tables 4, 5, and 6, respectively, the amounts of *α*-linolenic acid (ALA) and DHA were higher in the tissues of rats fed on the omega-3 enriched chevon diet compared to those on the CON diet. The *α*-linolenic acid in the omega-3 enriched chevon group was significantly (*P* < 0.05) higher than in the rat muscle tissue, liver, and heart tissues of the CON group. The amount of ALA was also higher in the hearts of the omega-3 enriched chevon fed rats (11.43/100 g tissue) compared to the CON rats (4.04 mg/100 g tissue). The trend was the same for the liver of the omega-3 enriched chevon diet group (22.85 mg/100 g tissue) compared to the CON group (9.52 mg/100 g tissue). The total n-3 PUFA in the rat muscle tissues of the omega-3 enriched chevon group was significantly (*P* < 0.05) higher (79.42 ± 7.57) compared to the CON group (55.56 ± 5.31). The n-6 : n-3 FA ratios (FAR) in the muscle tissue of the omega-3 enriched chevon group were significantly (*P* < 0.05) lower (4.46 ± 0.42) compared to the CON group (5.28 ± 0.31) (Table 4).

### 3.4. Liver Tissue Gene Expression

**Figure 1:**
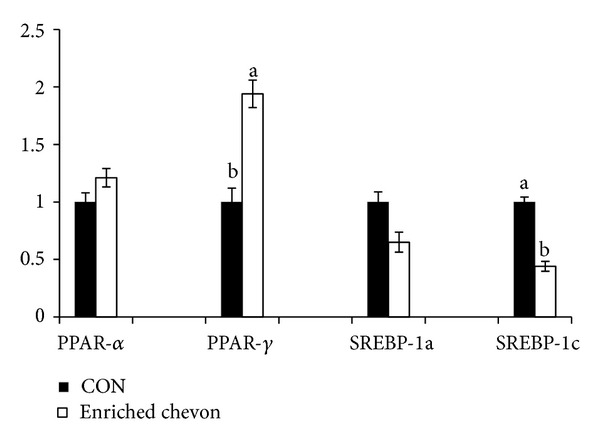
Comparison of relative gene expression in the liver tissue of rats fed on either control or omega-3 enriched chevon diets. Values were normalized with a housekeeping gene, *β*-actin. Then, treated samples were expressed relative to the gene expression of the CON group. Values are means ± 1 standard error bar. Values with different superscripts differ significantly at *P* < 0.05.

The relative expression of the genes in the liver tissues of the rats in the omega-3 enriched chevon group compared to CON group is shown in Figure 1. The PPAR-*α* and SREBP-1a genes showed a similar level of expression in both treatment groups (*P* > 0.05) indicating that the omega-3 enriched chevon diet had no effect on the gene expression of the PPAR-*α* and SREBP-1a. However, the omega-3 enriched chevon diet altered the PPAR-*γ* and SREBP1c expression in the rat liver where the increased omega-3 FA upregulated the PPAR-*γ* expression while the SREBP-1c expression was downregulated significantly (*P* < 0.05) in the omega-3 enriched chevon group compared to the CON group.

## 4. Discussion

Several studies have reported the cholesterol lowering effect of diets containing high *α*-linolenic acids [16, 17]. The plasma cholesterol level in the CON group (79 mg/dL) was in the normal range of rats as reported by other researchers for example Bansode et al. [18] who fed thier rats with peanut skin polyphenol (74.00 mg/dL) and Tong et al. [19] who fed their rats with oat oil (77.34 mg/dL). Various cardioprotective effects of high dietary ALA have also been reported in both animal and human models [16, 17, 20]. The ALA rich diets have been shown to have beneficial effects on hepatic cholesterol metabolism in rats fed high fat [17]. The aim of the present study was to bridge this gap and to compare the effects of ALA enriched meat on FA and gene expression of the hepatic cell. One of the objectives was to bring out the differences in pathways through which the ALA enriched meat may exert their beneficial effects on lipid metabolism.

### 4.1. Rat Muscle Tissue, Liver, and Heart Fatty Acids

These results suggest that eating enriched chevon with omega-3 FA increases the amount of the omega-3 FA in the muscle tissue, heart, and liver of rats. The ratio of dietary n-6 : n-3 was parallel to what Devarshi et al. [21] reported in their study using rat subjects which has a positive impact upon health indicators. Our findings also suggest a statistically significant increase in the ALA content of the rat muscle tissue, liver, and heart as a result of feeding on the omega-3 enriched chevon diet. Other longer chain omega-3 FAs especially DHA also increased in the omega-3 enriched chevron group compared to CON group which supported the observation by Medeiros et al. [22] who fed rats with high omega-3 beef. The rats fed on the omega-3 enriched chevon diet not only ate more ALA than the CON rats but also consumed more EPA, docosapentanoic acid, and DHA. However it remains unsure if the higher levels of the longer chain omega-3 FAs in the rat tissues were because of the diet as the ALA levels were 3.65-fold and DHA were 1.99-fold higher in the omega-3 enriched chevon diet compared to the control.

The lower plasma cholesterol levels in the enriched chevon diets could be likely due to the higher omega-3 FA in that diet compared to the CON diet. These results imply a protective role of omega-3 FAs from ALA sources, including that from enriched chevon.

Tables 3 and 4 showed that DHA increased significantly in the muscle tissue and livers of the rats fed on the omega-3 enriched chevon diet. This could be due to the higher levels of EPA and DHA in the omega-3 enriched chevon diet and a conversion of ALA to DHA, which was also reported by Medeiros et al. [22] who fed rats with enriched omega-3 beef. It is generally accepted that, in vertebrates, the omega-3 and omega-6 PUFA compete for the Δ-6 desaturase enzyme in order to be converted into long-chain PUFA. Most of mammals, except for carnivores, can convert LA to AA and ALA to EPA and DHA, but it is a slow process. There is competition between omega-6 and omega-3 fatty acids for the desaturation enzymes. However, both Δ-5 and Δ-6 desaturases prefer omega-3 to omega-6 fatty acids [10].

The level of total omega-3 FAs in the chevon per 100 g was 27.11 mg for the CON chevon group and 51.11 mg for the enriched chevon group. Using these values, two 100 g servings of chevon per day would supply 102.22 mg of omega-3 FAs daily. People with heart diseases are recommended to consume 1 g of a combination of EPA and DHA per day [23]. Ng et al. [24] reported that the omega-6 to omega-3 FA ratio in Malaysians is about 20:1, which is way above the World Health Organization recommended ratio of 5–10 : 1. In view of the fact that local Malaysian foods are not good sources of ALA, EPA, or DHA, the greater consumption of omega-3 enriched foods such as enriched meat, milk, and dairy products or even omega-3 supplementation during pregnancy and lactation can reduce the omega-6 to omega-3 FA ratio [24].

### 4.2. Genes Involved in Fatty Acid Metabolism

The hepatic expression of the genes studied in this report is depicted in Figure 1. The PPAR-*α*, a member of the nuclear receptor family of PPARs, acts as a transcription factor and is found to be the key regulators of lipid and carbohydrate metabolism [25]. The PPAR-*α* and *γ* are activators of mitochondrial and peroxisomal FA *β*-oxidation in the liver. The PPAR-*γ* is an activator of FA synthesis and storage and PPAR-*β* is a regulator of FA oxidation in muscles which play different roles in metabolism [25]. In addition, PPAR-*γ* has beneficial effects on cholesterol levels [26]. Previously, it has been shown that omega-3 FAs protect against high-fat-induced hepatic insulin resistance and reduce triglyceride (TG) via a PPARs gene regulation [27]. Similarly, in our study, the ALA rich diet upregulated hepatic PPAR-*γ* along with a decrease in cholesterol levels in the omega-3 enriched chevon group. Thus, it seems that these effects of ALA rich diet are mediated by PPAR-*γ*. The *α*-linolenic acid seems to have a “cholesterol lowering effect” that is independent of PPAR-*α*. The PPAR-*γ* may not be responsible for the increase in the omega-3 fatty acids content of the rat tissues and the higher level of omega-3 fatty acids in the rat tissues was probably due to their higher levels in the diet which led to a higher tissue incorporation of the omega-3 fatty acids. The SREBPs are transcription factors involved in the regulation of FA and cholesterol metabolism in the liver [28]. Out of the two isoforms of SREBP-1, namely, 1a and 1c, SREBP-1c is relatively abundant in the liver. The upregulation of SREBP-1a has been shown to result in the increased production of TG and cholesterol [29], while upregulation of SREBP-1c increased the production of TG [30]. Several studies demonstrated that omega-3 FAs bring about their hypotriglyceridemic effects by lowering SREBP-1c expression [31–33]. Similarly, in our study, the ALA rich diet produced a correlational decrease in plasma cholesterol levels and downregulation of hepatic SREBP-1c in the rat liver (Figure 1). The ALA rich diet demonstrated the activation of PPAR-*γ* on one hand, which would enhance *β*-oxidation and on the other hand suppression of SREBP-1c, which would reduce lipogenesis. On the contrary, ALA rich diets had no effect on PPAR-*α* but reduced SREBP-1c.

## 5. Conclusions

In conclusion, the ALA rich diet showed a cholesterol lowering effect in the rat plasma. This study was the first to look at the effects of eating a high-ALA diet of enriched chevon and the impact on long-chain omega-3 FA composition of EPA and DHA in the FA of the muscle tissue, liver, and heart using a rat model. The results show that an increase in DHA occurs in the muscle tissue and liver of rats fed a high-ALA diet of enriched omega-3 chevon. Changing a part of the goat's diet can increase the omega-3 concentrations in the finished chevon which in turn increased the tissue omega-3 fatty acid concentrations and decreased plasma cholesterol when fed to rats; the latter correlated with an upregulation of PPAR-*γ* and a downregulation of SREBP-1c gene expressions.

## Figures and Tables

**Table 1:** Nutrient composition of the experimental diets.

Dietary treatments	CON	Omega-3 enriched chevon
Moisture%	18.60	17.60
Crude protein%	42.79	44.21
Crude fat%	16.05	14.97
Crude fiber%	3.13	2.83
Gross energy Kcal/g	4.66	4.60

**Table 2:** Names and sequences of the primers used in this study.

Gene	Forward (5′ to 3′)	Reverse (5′ to 3′)
PPAR-*α*	CGACAAGTGTGATCGAAGCTGCAAG	GTTGAAGTTCTTCAGGTAGGCTTC
PPAR-*γ*	GCGGAGATCTCCAGTGATATC	TCA GCGACTGGGACTTTTCT
SREBP-1a	ACACAGCGGTTTTGAACGACATC	ACGGACGGGTACATCTTTACAG
SREBP-1c	GGA GCC ATG GAT TGC ACA TT	AGG AAG GCT TCC AGA GAG GA
*β*-actin	CAGGAGATGGCCACTGCCGCA	CTCCTTCTGCATCCTGTCAGCA

**Table 3:** Fatty acid concentration (mg/100 g feed) of experimental diets (*n* = 6).

Fatty acid	CON	Enriched chevon
Palmitic acid (C16:0)	4329.05	4040.41
Heptadecanoic acid (C17:0)	47.39	115.31
Cis Heptadecanoic acid (C17:1)	36.23	136.24
Stearic acid (C18:0)	3219.66	4173.26
Oleic acid (C18:1n-9)	6937.77	5989.9
Vaccenic acid (C18:1trans-11)	12.12	30.72
Linoleic acid (C18:2n-6)	1399.84	1351.1
cis-9, trans-11 conjugated linoleic acid	10.51	14.76
cis-12, trans-10 conjugated linoleic acid	3.18	4.68
*α*-Linolenic acid (C18:3n-3)	16.13^a^	58.81^b^
Arachidic acid (C20:0)	103.48	96.45
Arachidonic acid (C20:4n-6)	110.75	74.92
Eicosapentaenoic acid (C20:5n-3)	5.21^a^	10.12^b^
Docosapentaenoic acid (C22:5n-3)	3.65	7.98
Docosahexaenoic acid (C22:6n-3)	2.12^a^	4.21^b^
^ 1^Total SFA	7699.58	8425.43
^ 2^Total n-6 PUFA	1510.59	1426.02
^ 3^Total n-3 PUFA	27.11^a^	81.11^b^
^ 4^n-6:n-3 FAR	55.721	17.58

^1^Total SFA = sum of C16:0 + C17:0 + C18:0 + C20:0.

^2^Total n-6 PUFA = sum of 18:2n-6 + 20:4n-6.

^3^Total n-3 PUFA = sum of C18:3n-3 + C20:5n-3 + C22:5 n-3 + C22:6n-3.

^4^n-6:n-3 FAR = (C18:2n-6 + C20:4n-6) ÷ (C18:3n-3 + C20:5n-3 + C22:5n-3 + C22:6n-3).

^a,b^Values with different superscripts between rows differ significantly at *P* < 0.05.

**Table 4:** Fatty acid concentration (mg/100 g tissue) in muscle tissue of rats fed either control or omega-3 enriched chevon diets (mean ± SE, *n* = 10).

Fatty acid	CON	Enriched chevon
Lauric acid (C12:0)	3.18 ± 0.58	3.23 ± 0.66
Myristic acid (C14:0)	23.22 ± 2.45	16.62 ± 1.83
Pentadecanoic acid (C15:0)	8.88 ± 0.67	8.48 ± 1.24
Palmitic acid (C16:0)	508.97 ± 100.87	353.94 ± 49.91
Palmitoleic acid (C16:1)	33.45 ± 6.01	29.29 ± 6.48
Heptadecanoic acid (C17:0)	14.43 ± 2.81	8.61 ± 1.39
Cis Heptadecenoic acid (C17:1)	11.04 ± 1.29	10.41 ± 2.01
Stearic acid (C18:0)	340.39 ± 50.70	286.13 ± 36.30
Oleic acid (C18:1n-9)	791.84 ± 123.37	844.81 ± 84.29
Linoleic acid (C18:2n-6)	199.43 ± 19.12	244.08 ± 34.06
*α*-Linolenic acid (C18:3n-3)	3.90 ± 0.37^a^	8.23 ± 1.93^b^
Arachidonic acid (C20:4n-6)	93.75 ± 7.11	110.38 ± 10.29
Eicosapentaenoic acid (C20:5n-3)	9.51 ± 1.31	12.26 ± 0.92
Docosapentaenoic acid (C22:5n-3)	7.83 ± 0.87	9.54 ± 0.91
Docosahexanoic acid (C22:6n-3)	34.32 ± 2.76^a^	49.39 ± 33.81^b^
^ 1^Total n-6 PUFA	293.18 ± 26.23	354.46 ± 24.35
^ 2^Total n-3 PUFA	55.56 ± 5.31^a^	79.42 ± 7.57^b^
^ 3^n-6:n-3 FAR	5.28 ± 0.31^a^	4.46 ± 0.42^b^

^1^Total n-6 PUFA = sum of 18:2n-6 + 20:4n-6.

^2^Total n-3 PUFA = sum of C18:3n-3 + C20:5n-3 + C22:5n-3 + C22:6n-3.

^3^n-6:n-3 FAR = (C18:2n-6 + C20:4n-6) ÷ (C18:3n-3 + C20:5n-3 + C22:5n-3 + C22:6n-3).

^a,b^Values with different superscripts between rows differ significantly at *P* < 0.05.

**Table 5:** Fatty acid concentration (mg/100 g tissue) in liver of rats fed either control or omega-3 enriched chevon diets (mean ± SE, *n* = 10).

Fatty acid	CON	Enriched chevon
Lauric acid (C12:0)	2.77 ± 0.31^a^	2.22 ± 0.12^b^
Myristic acid (C14:0)	26.75 ± 4.66^a^	23.55 ± 1.22^b^
Myristoleic acid (C14:1)	3.74 ± 0.52	2.85 ± 0.42
Pentadecanoic acid (C15:0)	10.00 ± 1.01^a^	7.33 ± 0.39^b^
Cis Pentadecanoic acid (C15:1)	4.14 ± 0.36^a^	3.56 ± 0.15^b^
Palmitic acid (C16:0)	599.36 ± 48.67	554.17 ± 28.76
Palmitoleic acid (C16:1)	31.21 ± 4.84	28.20 ± 1.11
Heptadecanoic acid (C17:0)	20.08 ± 2.64^a^	15.71 ± 0.95^b^
Cis Heptadecenoic acid (C17:1)	4.65 ± 0.43	4.63 ± 0.38
Stearic acid (C18:0)	853.33 ± 44.03	844.42 ± 50.04
Oleic acid (C18:1n-9)	1027.99 ± 75.86	962.26 ± 40.92
Linoleic acid (C18:2n-6)	486.42 ± 20.83	497.17 ± 20.37
*α*-Linolenic acid (C18:3n-3)	9.52 ± 0.59^a^	22.85 ± 0.89^b^
Arachidonic acid (C20:4n-6)	339.37 ± 16.58	364.97 ± 16.55
Eicosapentaenoic acid (C20:5n-3)	12.60 ± 1.05^a^	26.64 ± 0.93^b^
Docosapentaenoic acid (C22:5n-3)	20.27 ± 3.06^a^	45.48 ± 2.00
Docosahexanoic acid (C22:6n-3)	40.27 ± 3.06^a^	55.48 ± 2.00^b^
^ 1^Total n-6 PUFA	825.79 ± 37.41	862.14 ± 36.92
^ 2^Total n-3 PUFA	82.66 ± 5.76^a^	150.45 ± 9.82^b^
^ 3^n-6:n-3 FAR	9.99 ± 1.42	5.73 ± 1.34

^1^Total n-6 PUFA = sum of 18:2n-6 + 20:4n-6.

^2^Total n-3 PUFA = sum of C18:3n-3 + C20:5n-3 + C22:5n-3 + C22:6n-3.

^3^n-6:n-3 FAR = (C18:2n-6 + C20:4n-6) ÷ (C18:3n-3 + C20:5n-3 + C22:5n-3 + C22:6n-3).

^a,b^Values with different superscripts between rows differ significantly at *P* < 0.05.

**Table 6:** Fatty acid concentration (mg/100 g tissue) in heart of rats fed either control or omega-3 enriched chevon diets (mean ± SE, *n* = 10).

Fatty acid	CON	Enriched chevon
Lauric acid (C12:0)	3.18 ± 0.58	3.23 ± 0.66
Myristic acid (C14:0)	16.88 ± 4.13	12.61 ± 1.22
Myristoleic acid (C14:1)	37.28 ± 2.65	34.43 ± 1.50
Pentadecanoic acid (C15:0)	8.88 ± 0.67	8.48 ± 1.24
Cis Pentadecanoic acid (C15:1)	44.80 ± 3.68	50.85 ± 5.04
Palmitic acid (C16:0)	341.54 ± 39.49	269.39 ± 36.91
Palmitoleic acid (C16:1)	15.36 ± 2.76	16.25 ± 1.87
Heptadecanoic acid (C17:0)	14.27 ± 1.91	13.40 ± 1.52
Cis Heptadecenoic acid (C17:1)	20.16 ± 3.24	18.66 ± 0.93
Stearic acid (C18:0)	323.86 ± 44.36	280.50 ± 28.39
Oleic acid (C18:1n-9)	388.23 ± 43.20	425.42 ± 52.91
Linoleic acid (C18:2n-6)	306.98 ± 33.90	357.93 ± 31.19
*α*-Linolenic acid (C18:3n-3)	4.04 ± 0.74^a^	11.43 ± 1.63^b^
Arachidonic acid (C20:4n-6)	299.62 ± 32.19	305.47 ± 26.23
Eicosapentaenoic acid (C20:5n-3)	17.8 ± 2.62	29.5 ± 1.63
Docosapentaenoic acid (C22:5n-3)	7.8 ± 1.31	19.5 ± 0.97
Docosahexanoic acid (C22:6n-3)	62.8 ± 6.41	80.43 ± 5.32
^ 1^Total n-6 PUFA	606.6 ± 66.09	663.4 ± 57.42
^ 2^Total n-3 PUFA	92.44 ± 8.08^a^	140.86 ± 9.55^b^
^ 3^n-6:n-3 FAR	6.57 ± 0.61^a^	4.70 ± 0.16^b^

^1^Total n-6 PUFA = sum of 18:2n-6 + 20:4n-6.

^2^Total n-3 PUFA = sum of C18:3n-3 + C20:5n-3 + C22:5n-3 + C22:6n-3.

^3^n-6:n-3 FAR = (C18:2n-6 + C20:4n-6) ÷ (C18:3n-3 + C20:5n-3 + C22:5n-3 + C22:6n-3).

^a,b^Values with different superscripts between rows differ significantly at *P* < 0.05.
